# Prevalence of frailty and prediction of mortality in Chinese cancer patients using a frailty index‐based clinical algorithm—A multicentre study

**DOI:** 10.1002/cam4.4155

**Published:** 2021-07-28

**Authors:** Xi Jin, Yue Ren, Li Shao, Zengqing Guo, Chang Wang, Ying He, Lan Zhou, Minghua Cong, Hu Ma, Wei Wang, Chunling Zhou, Yongdong Feng, Yi Ba, Jianguo Gao, Miaomiao Lu, Mengmeng Zhang, Xue‐wei Gu, Chunhua Song, Hongxia Xu, Hanping Shi

**Affiliations:** ^1^ Department of Gastroenterology The First Affiliated Hospital, School of Medicine Zhejiang University Hangzhou China; ^2^ Institute of Translational Medicine The Affiliated Hospital of Hangzhou Normal University Hangzhou China; ^3^ Department of Medical Oncology Fujian Cancer Hospital Fujian Medical University Cancer Hospital Fuzhou China; ^4^ Cancer Center of the First Hospital of Jilin University Changchun China; ^5^ Department of Clinical Nutrition Chongqing General Hospital Chongqing China; ^6^ Department of Nutrition Third Affiliated Hospital of Kunming Medical College Tumor Hospital of Yunnan Province Kunming China; ^7^ Comprehensive Oncology Department Cancer Hospital Chinese Academy of Medical Sciences Beijing China; ^8^ Department of Oncology Affiliated Hospital of Zunyi Medical University Zunyi China; ^9^ Cancer Center The First People's Hospital of Foshan Foshan China; ^10^ Department of Clinical Nutrition The Fourth Affiliated Hospital Harbin Medical University Harbin China; ^11^ Department of Surgery Tongji Hospital, Tongji Medical College Huazhong University of Science and Technology Wuhan China; ^12^ Department of Gastrointestinal Oncology Tianjin Medical University Cancer Institute and Hospital National Clinical Research Center for Cancer Tianjin China; ^13^ Department of Epidemiology and Statistics College of Public Health Zhengzhou University Zhengzhou China; ^14^ Department of Nutrition, Department of Nutrition Daping Hospital & Research Institute of Surgery Third Military Medical University Chongqing China; ^15^ Department of Gastrointestinal Surgery/Department of Clinical Nutrition Beijing Shijitan Hospital, Capital Medical University/The 9th Clinical Medical College Peking University Beijing China

**Keywords:** blood test, cancer, frailty, mortality

## Abstract

**Purpose:**

To investigate the frailty status in Chinese cancer patients through establishing a novel prediction algorithm.

**Methods:**

The percentage of frailty in various age groups, locations, and tumor types in Chinese cancer patients was investigated. The prediction capacity of frailty on mortality of Chinese cancer patients was analysed by the frailty index composing of routine laboratory data (FI‐LAB) accessible from a blood test and calculated as the ratio of abnormal factors to 22 total variables. The establishment of a novel algorithm, MCP (mortality of cancer patients), to predict the 5‐year mortality in Chinese cancer patients was accomplished and the algorithm's prediction capacity was tested in the training and validation sets using receiver operating characteristic (ROC) analysis.

**Results:**

We found that the risk of death in cancer patients can be successfully identified through FI‐LAB. The univariable and multivariable Cox regression were used to evaluate the effect of frailty on death. In the 5‐year follow‐up, 20.6% of the 2959 participants (age = 55.8 ± 11.7 years; 43.5% female) died, while the mean FI‐LAB score in baseline was 0.23 (standard deviation = 0.13; range = 0–0.73). Frailty (after adjusting for gender, age, and other confounders) directly correlated with an increased risk of death, hazard ratio of 12.67 (95% confidence interval [CI]: 7.19, 22.31), compared to those without frailty. In addition, the MCP algorithm (MCP) = 3.678 × FI‐LAB + 1.575 × sex + 1.779 × first tumor node metastasis staging, presented an area under the ROC (AUC) of 0.691 (95% CI: 0.656–0.726) and 0.648 (95% CI: 0.613–0.684) in the training and validation sets, respectively.

**Conclusion:**

Frailty as defined by FI‐LAB was common and indicated a significant death risk in cancer patients. Our novel developed algorithm MCP had a passable prediction capacity on 5‐year MCP.

## Graphical abstract

To investigate the frailty status in Chinese cancer patients through establishing a novel prediction algorithm. Frailty as defined by FI‐LAB (frailty index composing of routine laboratory data) was common and indicated a significant death risk in cancer patients. Our novel developed algorithm mortality of cancer patients had a passable prediction capacity on 5‐year mortality of cancer patients.

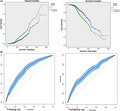

## 1 BACKGROUND

Frailty, an emerging global health burden, carries major implications in clinical practice and public health. Considering the exponential growth of the ageing population, prevalence of frailty will also undoubtedly rise.^1^ Frailty is characterised by a decline in function across multiple physiological systems, with an increased vulnerability to stressors, especially in those with cancer.^2^ The concept of frailty is progressively being used in primary, acute, and specialist care, but in the last three decades, efforts establish a standard procedure to identify frailty have been futile, however, three significant factors have remained consistent.^3^ First, frailty is multidimensional, with both physical and psychosocial factors involved in its development. Second, although its prevalence increases with age, frailty is an extreme consequence of the normal ageing process. Third, frailty is a dynamic and fluctuating status.^3^ Subjects with frailty are not only at an increased risk of adverse outcomes, including accidents like falling, hospitalisation, and mortality,^4^ but also carry individual burdens such as an impaired quality of life and loneliness.^5^ Prevalence of cancer and resultant mortality has been steadily increasing over many decades, subsequently becoming the leading cause of death in China since 2010.^6^, ^7^, ^8^ Because signs such as malnutrition and cachexia are often observed during the end stages of cancer, it is plausible to assume possible involvement of frailty. Although the association between cancer and frailty has previously been summarised,^2^, ^9^ these studies mainly focus on the elderly, whereas frailty status in Chinese cancer patients and its prediction capacity in cancer mortality have seldom been reported and, therefore, warrants further investigation.

Currently, a major obstacle in the study of frailty is the lack of global standardised assessment.^10^ The most widely used methods are based on frailty physical phenotype and frailty index, with various modifications described in different studies.^11^ The physical phenotype, being more subjective, is usually applied in cohort studies,^12^ whereas the frailty index is a more objective score based on cumulative health‐related variables.^13^ The frailty index is defined as the number of variables (out of the normal range of investigated parameters) observed in a patient divided by the sum of all investigated parameters. For example, if 20 deficits are reviewed out of which only 2 deficits are found, the frailty index would be 0.01 (2/20). Therefore, range of the frailty index lies between 0 and 1, with a score approaching 1 suggesting a greater degree of frailty. In hospitalised cancer patients, the frailty index appears to be a strong predictor of adverse clinical outcomes.^14^ However, creation of a frailty index in a busy clinical setting is time‐consuming. This can be circumvented by generating a frailty index that depends on routinely collected clinical data. FI‐LAB (frailty index composing of routine laboratory data) was developed from the Canadian Study of Health and Aging (CSHA) cohort based on 21 laboratory variables in older patents for death risk prediction.^15^ Several other studies have confirmed that FI‐LAB is feasible, valid, and closely associated with frailty indices based on complex, self‐reported data for the prediction of mortality.^16^, ^17^ However, these studies only include Caucasian individuals aged from 35 to 89 years old and its association in other stages of life and in cancer patients have yet to be elucidated.

Hence, in this study, we carried out a multicentre, prospective, observational study based on the largest, Chinese, prospective oncology and nutrition study (Investigation on Nutrition Status and its Clinical Outcome of Common Cancers [INSCOC] study) to investigate the frailty status of Chinese patients with varying cancer types residing in different provinces using the FI‐LAB, established only on routine laboratory data. The FI‐LAB was also used to predict patient mortality. In addition, we successfully devised a risk assessment algorithm, MCP (mortality of cancer patients) comprising of FI‐LAB and other additional factors to predict mortality in Chinese cancer patients.

## 2 METHODS

### 2.1 Participants and ethics

In 2020, we collected data from the Chinese Society of Nutritional Oncology initiated clinical research project known as the INSCOC starting from 2013. This observational, multi‐centre, and hospital‐based prospective cohort study was registered with the Chinese Clinical Trial Registry and approved by the local ethical committees of all the participant's respected hospitals, with a formal written consent taken from every patient. This study was carried out in accordance with the declaration of Helsinki. The primary outcome was the prevalence of frailty in Chinese patients with local regional, recurrent, or metastatic cancer of all ages. The secondary outcome included prediction capacity of nutrition status, physical performance, and quality of life at admission on overall survival. Moreover, we studied the association between frailty and various demographic characteristics, and subsequently established a mortality prediction model based on FI‐LAB.

All patients were recruited by clinical investigators in various departments of participating hospitals. The patient diagnosis at time of recruitment included 1 of the following 18 malignancies: lung cancer, gastric cancer, liver cancer, colorectal cancer, breast cancer, esophageal cancer, cervical cancer, endometrial cancer, nasopharyngeal carcinoma, malignant lymphoma, leukemia, pancreatic cancer, ovarian cancer, prostate cancer, bladder cancer, brain tumor, biliary tract malignant tumor, and gastric stromal tumor. Anatomical site description in the medical records was in accordance with the 10th edition of the International Classification of Disease (ICD‐10). The inclusion criteria are as follows: >13 years of age, tumor diagnosed by pathology and staged as local, metastatic, and/or loco‐regional relapse, with the patient having a clear understanding of their disease. The exclusion criteria are as follows: organ transplantation, pregnant women, concurrent diagnosis of human immuno‐deficiency virus infection, intensive care unit admission at the beginning of recruitment, and hospitalisation more than two times during the investigation.

Several co‐existing diseases possibly affecting the frailty status were investigated at time of admission and included hepatic cirrhosis, chronic hepatitis, chronic pancreatitis, stroke, chronic obstructive pulmonary disease, myocardial infarction, diabetes, hypertension, coronary heart disease, anemia, hyperthyroidism, hypothyroidism, chronic nephrosis, dialysis treatment, osteoporosis, ulcerative colitis, Crohn's disease, chronic diseases of the biliary system, systemic lupus erythematosus, and tuberculosis. For this analysis, participants lacking follow‐up data (22 cases) and lacking age data (4 cases) were excluded, bringing the final sample size to be 2959 (male 1673; female 1286).

### 2.2 Frailty index FI‐LAB establishment based on lab variables

In this study, frailty was defined by FI‐LAB. We constructed FI‐LAB based on 22 lab variables measured from a fasting blood sample. Variables were selected according to previous studies,^15^, ^18^ including white blood cell count, neutrophils, platelets (PLT), hematocrit, red blood cells, hemoglobin, mean corpuscular volume, mean corpuscular hemoglobin, mean corpuscular hemoglobin concentrations (MCV, MCH, and MCHC, respectively), blood glucose, total and direct bilirubin (TBil and DBil, respectively), alanine transaminase (ALT), albumin (Alb), globulin (Glob), urea, creatinine (CREA), uric acid (URIC), cholesterol (CHOL), high‐density and low‐density lipoprotein cholesterol (HDL‐C and LDL‐C, respectively), and triglycerides (TG). Each variable was labeled as either 1 or 0, with 1 indicating that the values exceeded the normal range or cutoff, and 0 indicating that the values were within the normal range.^19^ FI‐LAB is defined as the sum of all existing variables from parameter divided by the sum of all considered parameters (here, 22). Theoretically, FI‐LAB is a continuous variable between 0 and 1 for each given individual. In this study, established FI‐LAB cutoff points (0.21) were employed according to previous studies.^18^, ^19^

### 2.3 Data for mortality and other co‐variables

Mortality data were collected till 2020 for all patients. Patient status was defined as survival or death, and loss of follow‐up was defined as censored data. The time of death and co‐variables were recorded, including the individual's age, gender, and chronic disease using a general questionnaire through direct interview by well trained volunteers. All reported chronic illnesses were diagnosed by certified local physicians.

### 2.4 Statistical analysis

Descriptive statistics were used for baseline characteristics. Continuous or categorical variables were described using mean values, standard deviation (SD), numbers, or percentages and the differences between survival and frailty status (determined by FI‐LAB) were evaluated by applying the unpaired Student's *t*‐test for continuous variables, and chi‐square test for categorical variables. We applied Cox proportional hazard regression models to determine the hazard ration (HR) and its 95% confidence intervals (CIs) of frailty, with the function of increased mortality represented by each parameter in FI‐LAB and overall frailty status. Univariate and multivariate cox regression analyses were used to identify the predictors of mortality. Factors significantly associated with mortality in the univariate analysis (*p* < 0.05) were tested for multicollinearity using linear regression. Factors were included in the algorithm if they remained significant in the multivariate analysis. Using these variables, a Cox regression‐based nomogram was developed to predict mortality in cancer patients. The discriminative power of the model was then evaluated based on discrimination and calibration. The predictive accuracy of the nomogram was validated using receiver operating characteristic (ROC) and quantified by the area under the curve (AUC) and 95% CIs. An AUC of 0.5 and 1.0 indicate no relationship and perfect concordance while an AUC >0.75 is considered to have a relatively good discrimination. The SPSS version 22 (SPSS Inc.) and Prism 8 were applied for all statistical analyses and plots. The statistical significance was set as two‐tailed with *p* at <0.05.

## 3 RESULTS

### 3.1 Baseline characteristics of patients with various cancers and frailty

A total of 2959 patients (ranging from 13 to 94 years) with various cancers were included in this study, with a mean age of 55.8 ± 11.7 years and female percentage of 43.5%. The participant's median, mean, and maximum scores of FI‐LAB were 0.227, 0.233, and 0.730, respectively, with 99th percentile score being 0.591. The overall prevalence of frailty was 55.2% (FI‐LAB ≧ 0.21; 95% CI 53.4–57.0). With regards to gender distribution, men had higher FI‐LAB score compared to women (0.24 ± 0.13 vs. 0.23 ± 0.13; *p* = 0.007). As shown in Table 1, cancer patients with frailty had a significantly older age, were male, and had increased levels of total cholesterol (TC), TG, and LDL‐C, as well as lower levels of HDL‐C, serum uric acid (SUA), and body mass index (BMI). Regarding co‐existing diseases, cancer patients with frailty had significantly higher rates of hypertension, diabetes, anemia, and chronic hepatitis. More importantly, the death rate is nearly doubled in cancer patients with frailty than those without frailty (26.0% vs. 14.3%, *p* < 0.001).

**TABLE 1:** Characteristics of the study population according to frailty assessed by FI‐LAB

	Frailty		*p* value
	No (*n* = 1352)	Yes (*n* = 1607)	
Age (years)	55.0 ± 11.9	56.5 ± 11.6	<0.001**
Male (%)	54.1	58.6	0.016*
BMI (kg/m^2^)	23.2 ± 3.3	22.8 ± 5.2	0.042*
Weight (kg)	62.0 ± 10.4	61.6 ± 15.4	0.319
Height (cm)	163.5 ± 7.8	164.0 ± 7.7	0.095
TG (mmol/L)	1.4 ± 1.0	1.6 ± 1.1	<0.001**
TC (mmol/L)	4.7±1.2	4.8 ± 1.6	0.012*
HDL‐C(mmol/L)	1.3±0.5	1.2 ± 0.5	<0.001**
LDL‐C (mmol/L)	2.9±0.8	3.1 ± 1.0	<0.001**
SUA (μmol/L)	314.6±73.0	306.3 ± 95.8	0.008*
Hypertension (%)	5.8	10.4	0.002*
Diabetes (%)	14.9	19.3	<0.001**
Cardiovascular disease	2.8	3.7	0.19
Anemia	0.5	2.2	<0.001**
Chronic hepatitis	4.5	6.2	0.048*
Death (%)	14.3	26.0	<0.001**

Note

Data represents the mean ± SD unless otherwise indicated.

Abbreviations: BMI, body mass index; FI‐LAB, frailty index composing of routine laboratory data; HDL‐C, high‐density lipoprotein cholesterol; LDL‐C, low‐density lipoprotein cholesterol; SD, standard deviation; SUA, serum uric acid; TC, total cholesterol; TG, triglycerides.

* *p* < 0.05

** *p* < 0.01.

### 3.2 Frailty investigation in cancer patients categorized by location, age, and tumor type

**FIGURE 1:**
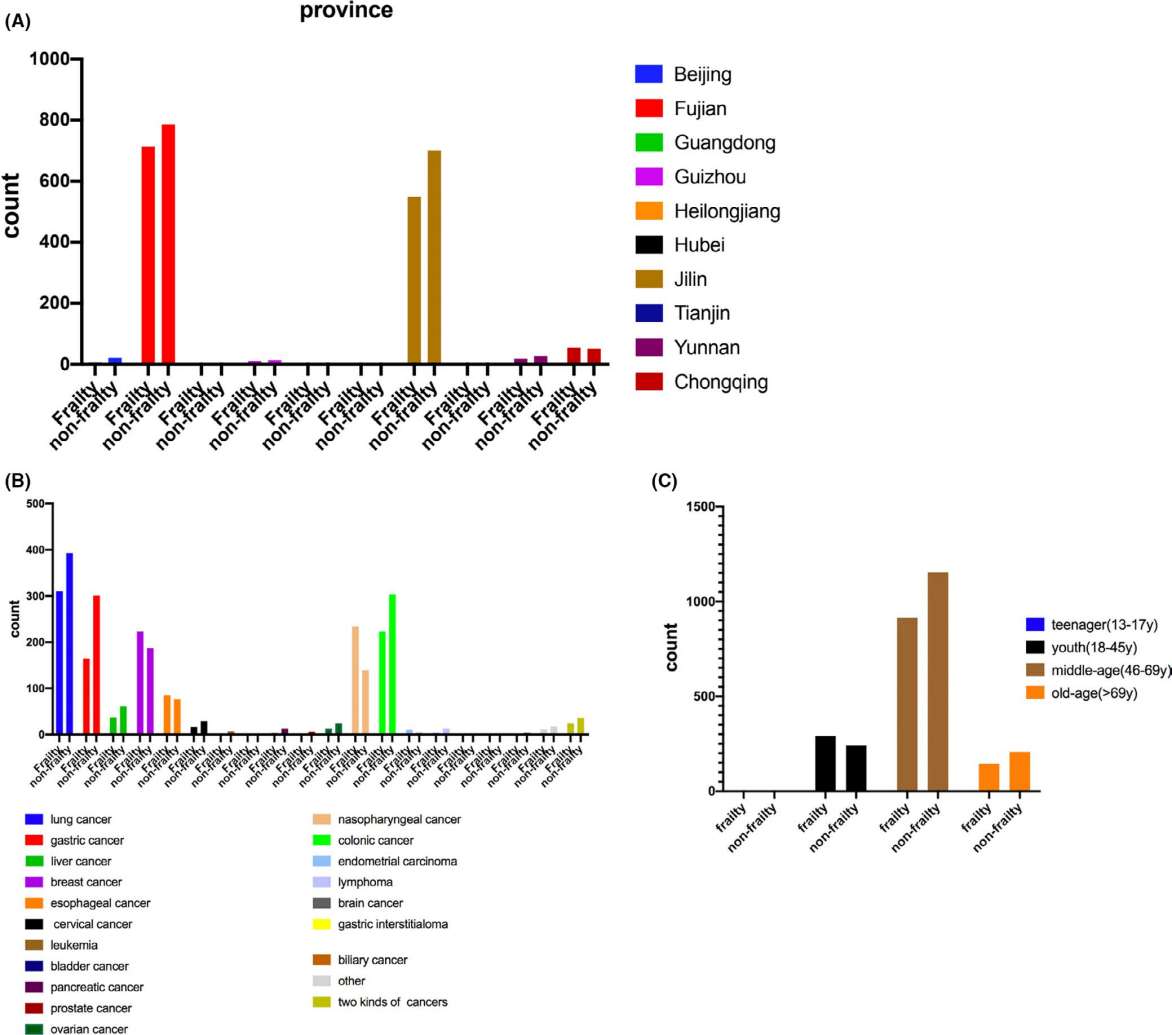
The number of cancer patients with/without frailty in different provinces (A), cancers (B), and ages (C)

We investigated the incidence rate of frailty in cancer patients categorized by location, age, and tumor type. The patients were mainly from Fujian province (total 1499, frailty incidence rate of 52.4%), Jilin province (total 1249, frailty incidence rate of 56.0%), and Chongqing city (total 105, frailty incidence rate of 48.6%), with no significant difference in frailty among them (*χ* ^2^ = 4.8, *p* = 0.09, Figure 1A). Frailty incidence among different cancers varied (Figure 1B), with participants with lung cancer at 55.9%, gastric cancer at 64.7%, liver cancer at 45.6%, breast cancer at 45.6%, esophageal cancer at 47.2%, cervical cancer at 64.4%, nasopharyngeal cancer of 37.3%, and colonic cancer at 57.6% (*χ* ^2^ = 86.3, *p* < 0.001 among these groups). Frailty incidence also differed by age of the participants (Figure 1C), with teenagers (13–17 year) at 57.1%, the youth (18–45 year) at 45.4%, the middle‐age (46–69 year) at 55.8%, and the elderly (>69 year) at 59.0%. Chi‐square test revealed a statistically significant difference in frailty incidence between youth and middle‐age groups (*χ* ^2^ = 18.4, *p* < 0.001) and between youth and elderly groups (*χ* ^2^ = 15.6, *p* < 0.001).

### 3.3 Comparison between patients in death and survival groups

The all‐cause mortality of patients in this study was 20.6%. Subjects in the death group were significantly older and frailer than those in survival group (58.3 ± 11.5 vs. 55.0 ± 11.7, *p* < 0.001; 0.28 ± 0.14 vs. 0.21 ± 0.12, *p* < 0.001). The death group had a higher proportion of participants with frailty compared to the survival group (68.4% vs. 49.7%, *p* < 0.001). Those in the survival group had significantly higher TG, SUA, BMI, and HDL‐C levels than those in the death group (23.3 ± 4.9 vs. 22.4 ± 3.57, *p* < 0.001; 1.3 ± 0.5 vs. 1.2 ± 0.6, *p* < 0.001). Table 2 shows the attributes of subjects according to the status of survival.

**TABLE 2:** Characteristics of cancer patients in the survival and death groups

	Status of survival		*p* value
	Survival (*n* = 1918)	Death (*n* = 611)	
Age (years)	55.0 ± 11.7	58.3 ± 11.5	<0.001**
Male (%)	53.0	64.6	<0.001**
BMI (kg/m^2^)	23.3 ± 4.9	22.4 ± 3.5	<0.001**
Weight (kg)	62.1 ± 14.6	61.2 ± 11.2	0.147
Height (cm)	163.2 ± 7.8	165.2 ± 7.8	<0.001**
TG (mmol/L)	1.6 ± 1.1	1.4 ± 0.9	0.007*
TC (mmol/L)	4.8 ± 1.4	4.7 ± 1.6	0.095
HDL‐C (mmol/L)	1.3 ± 0.5	1.2 ± 0.6	<0.001**
LDL‐C (mmol/L)	3.0 ± 0.9	2.9 ± 0.9	0.123
SUA (μmol/L)	311.5 ± 81.7	301.3 ± 91.9	0.014*
Hypertension (%)	17.4	18.7	0.483
Diabetes (%)	8.0	9.3	0.312
Cardiovascular disease (%)	3.2	4.4	0.416
Anemia (%)	1.3	2.1	0.145
Chronic hepatitis (%)	5.5	5.7	0.85
Frailty (%)	49.7	68.4	<0.001**

Abbreviations: BMI, body mass index; HDL‐C, high‐density lipoprotein cholesterol; LDL‐C, low‐density lipoprotein cholesterol; SD, standard deviation; SUA, serum uric acid; TC, total cholesterol; TG, triglycerides.

* *p* < 0.05.

** *p* < 0.01.

### 3.4 Prediction capacity of FI‐LAB on a 5‐year mortality of cancer patients

**FIGURE 2:**
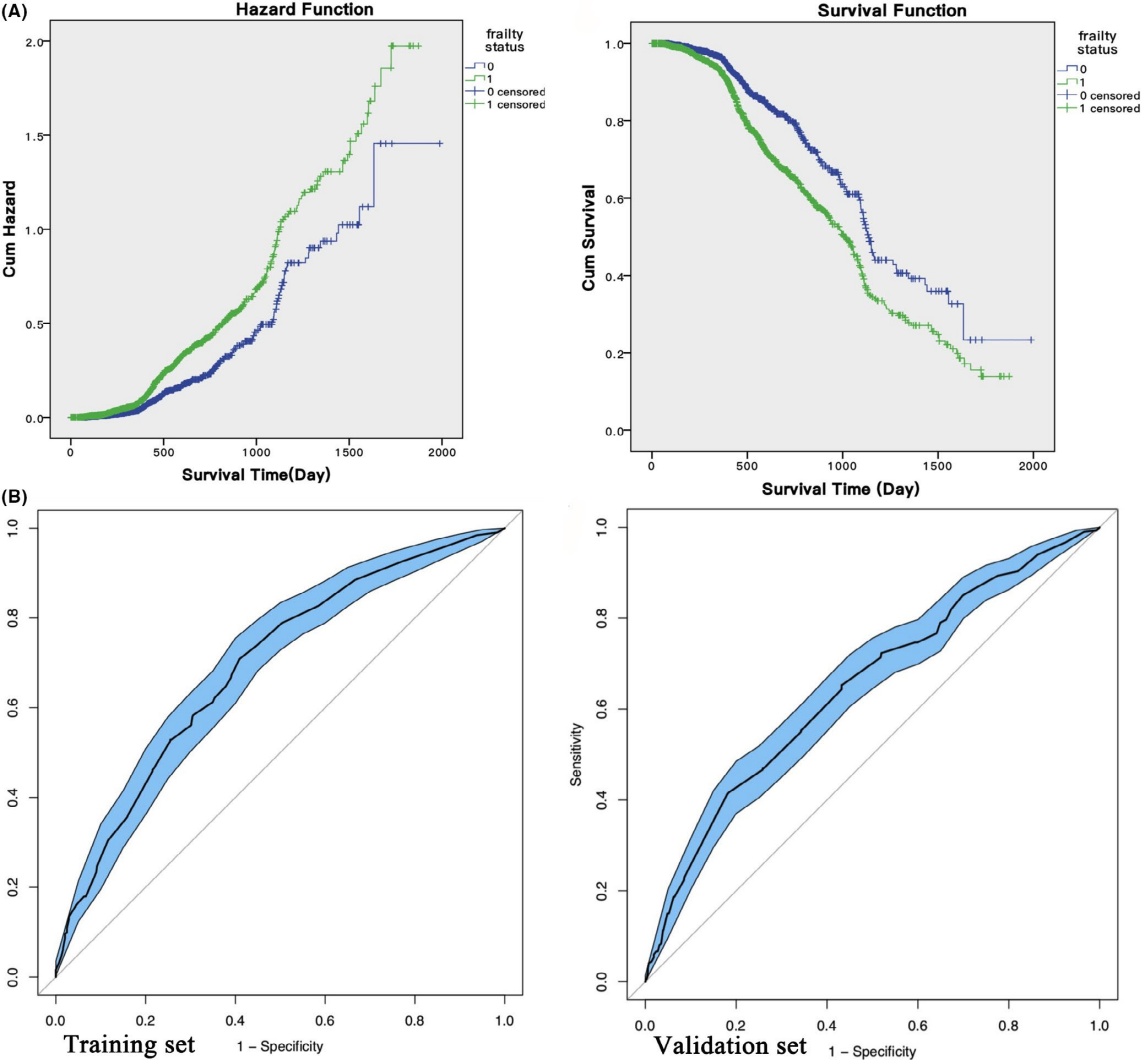
Effect of FI‐LAB and MCP in mortality prediction of patients with various cancers. (A), Cumulative hazard of death (left panel) and survival (right panel) in the study population, according to frailty at baseline. (B), The ROC curve of training (left panel) and validation (right panel) sets indicated passable predictive accuracy of MCP on mortality of patients with various cancers. 0 indicated no frailty; 1 indicated frailty. FI‐LAB, frailty index composing of routine laboratory data; MCP, mortality of cancer patients

Outcomes from the adjusted and unadjusted Cox regression models of frailty and mortality are presented in Table 3. Subjects with frailty had a significant higher increased risk of mortality compared with those without frailty (HR: 13.62, 95% CI: 7.77–23.87, *p* < 0.001). The Cox proportional hazard regression model was quite stable (HR: 12.67, 95% CI: 7.19–22.31) after compensating for age, gender, BMI, hypertension, cardiovascular disease, anemia, diabetes, and chronic hepatitis. The cumulative death hazard and survival of the study population based on FI‐LAB at baseline are presented in Figure 2A, where the median survival time of patients with frailty was significantly shorter than patients without frailty (1010 days vs. 1138 days, *χ* ^2^ of Log‐rank test = 33.2, *p* < 0.001; *χ* ^2^ of Breslow test = 32.9, *p* < 0.001 and *χ* ^2^ of Tarone‐Ware = 36.4, *p* < 0.001). Although statistical analysis showed that most variables (neutrophils, platelets, red blood cells, MCV, MCH, MCHC, blood glucose, TBil, DBil, Glob, URIC, and CHOL) comprising FI‐LAB did not affect the 5‐year mortality risk, the levels of PLT, CREA, ALT, and LDL‐C increased the 5‐year mortality risk, while hematocrit, hemoglobin, TG, HDL‐C, and Alb decreased the 5‐year mortality risk (Table S1).

**TABLE 3:** Estimation of the accuracy of FI‐LAB on mortality, modeled with Cox regression

	No frailty	Frailty HR (95% CI)
Unadjusted model	1 (Reference)	13.62 (7.77, 23.87)
Adjusted model 1^a^	1 (Reference)	12.20 (6.95, 21.45)
Adjusted model 2^b^	1 (Reference)	12.27 (6.97, 21.55)
Adjusted model 3^c^	1 (Reference)	12.67 (7.19, 22.31)

Abbreviations: BMI, body mass index; CI, confidence interval; FI‐LAB, frailty index composing of routine laboratory data; HR, hazard risk.

^a^ Adjusted for age, gender.

^b^ Adjusted for age, gender, BMI.

^c^ Adjusted for age, gender, BMI, hypertension, cardiovascular disease, anemia, diabetes, and chronic hepatitis.

### 3.5 Development of a novel mortality prediction algorithm based on FI‐LAB

Based on FI‐LAB and other independent factors associated with mortality, we developed an algorithm to predict mortality in cancer patients (shown in Table 4), MCP = 3.678 × FI‐LAB + 1.575 × sex + 1.779 × first TNM Staging (Tumor Node Metastasis). In this algorithm, FI‐LAB ranged from 0 to 1, sex was labeled as either 1 or 2, with 1 indicating man, and 2 indicating woman, first TNM staging is 0 or 1, with 0 indicating that there was no lymph node metastasis or distant organ metastasis, while 1 indicates at least one lymph node metastasis or distant organ metastasis. The higher the MCP value, the higher the mortality rate. Patients were randomly divided into either a training set (*n* = 966) or a validation set (*n* = 1963). A ROC curve and a calibration plot were applied to assess the predictive value of MCP. The training set had significantly less men, but more cardiovascular disease, anemia, and chronic hepatitis as well as higher BMI, weight, TG, and lower LDL‐C and SUA (Table S2). The MCP algorithm presented mortality prediction with acceptable accuracy, with an AUC of 0.691 (95% CI: 0.656–0.726) and 0.648 (95% CI: 0.613–0.684) in the training and validation sets, respectively (Figure 2B). In this algorithm, frailty index played the most important role in accordance with the hazard ratio of frailty (HR: 13.62, 95% CI: 7.77–23.87, *p* < 0.001).

**TABLE 4:** Univariate analysis of factors predicting mortality in cancer patients

Variables	Cox univariate analysis	
	*B*	*p*
Sex	−0.536	<0.001**
Age	0.020	<0.001**
Liver cirrhosis	−0.427	0.115
Chronic hepatitis	−0.257	0.141
Shock	−3.002	0.797
COPD	−0.370	0.603
Myocardial infarction	0.179	0.758
Diabetes	0.025	0.857
Hypertension	0.064	0.539
Cardiovascular disease	0.165	0.401
Anemia	0.159	0.572
Hyperthyroidism	−1.682	0.093
Hypothyroidism	−0.976	0.330
Chronic pancreatitis	0.910	0.199
Osteoporosis	−0.395	0.495
Ulcerative colitis	−1.212	0.087
Crohn disease	−3.002	0.771
Biliary disease	0.031	0.857
Chronic kidney disease	1.227	0.084
Tuberculosis	−3.001	0.832
Tumor family history	−0.415	0.002*
Surgery	−0.311	0.022
Chemotherapy	0.346	<0.001**
Radiotherapy	−0.598	<0.001**
Heat therapy	0.292	0.477
Targeted therapy	0.254	0.197
Immunotherapy	−0.330	0.078
Endocrine therapy	−1.195	0.017
Complication therapy	0.645	0.001*
Frailty index	2.622	<0.001**
Height	0.016	0.002*
Weight	−0.014	<0.001**
BMI	1.000	0.035*
Trauma	−1.294	0.010*
Gt 65 years	0.308	0.001*
Disease score	0.250	0.003*
Disease staging	0.323	<0.001**
Life quality score	0.028	<0.001**
Outcome after 30 days	1.372	<0.001**
Total hospital stay	−0.003	0.462
ICU stay	−0.398	0.011*
First TNM staging	0.558	<0.001**

Abbreviations: BMI, body mass index; COPD, chronic obstructive pulmonary disease; ICU, intensive care unit.

* *p* < 0.05

** *p* < 0.01.

## 4 DISCUSSION

In this study, we first investigated the frailty status of 2959 Chinese cancer patients and performed a subgroup analysis based on tumor type, age, and location, which is, to the best of our knowledge, the largest sample size in this specific population. A previous meta‐analysis on prevalence and outcomes of frailty in older cancer patients from 20 studies only included 2916 participants.^9^ Moreover, our study is the first to evaluate the prediction capacity of frailty index (FI‐LAB) on mortality in cancer patients. Finally, based on FI‐LAB and other independent laboratory parameters, we developed a novel algorithm, MCP, to predict the 5‐year MCP, and achieved an near passable ROC score of 0.691 and 0.648 in the training and validation sets, respectively. To summarize, we demonstrated frailty assessed by FI‐LAB was linked to an increased mortality risk, and our novel developed algorithm MCP had a passable prediction capacity on the 5‐year MCP, calling for more attention on the diagnosis and treatment of frailty in cancer patients.

We also found men were more susceptible to frailty than women, according to both FI‐LAB score and frailty incidence, differing from most previous studies showing higher frailty rate in women than in men using self‐reported frailty physical phenotype data.^20^, ^21^, ^22^ This phenomenon may be attributed to our enrollment of patients of all ages, whereas previous studies mainly focused on the elderly. However, one study employing laboratory parameters to constitute the frailty index also found a higher FI‐LAB score in men than women among older patients.^23^ Therefore, the methods used for assessing frailty may also contribute to this difference. In addition, it is well acknowledged that laboratory variables are more objective than health‐related deficits from self‐reported data,^24^ which include biologically interconnected symptoms and signs (unintentional weight loss, low handgrip strength, slow working speed, low physical activity level, and self‐reported exhaustion).^25^ It is implied that FI‐LAB showed a greater advantage in evaluating the effect of frailty on cancer mortality than other frailty assessment methods.

The prediction capacity of FI‐LAB on mortality was similar between our study and other studies that included participants aged from 35 to 89 years.^15^, ^16^ One study found that the association between FI‐LAB and mortality was not statistically significant among the 20–39 years age group,^16^ conflicting with our finding of a statistically significant association between youth and middle‐age groups as well as youth and elderly groups. Several studies report a direct relationship between age and risk of frailty,^26^, ^27^, ^28^ and incidence of frailty with old age is associated with increasing physiological dysregulation with ageing in a nonlinear manner. When frailty is present, response to a stressor becomes highly dysregulated with a higher likelihood of adverse outcomes.^25^, ^29^, ^30^ Weight loss has been incorporated in many frailty measures, and is postulated to be a modifiable factor in frailty.^31^, ^32^, ^33^ In our study, the frailty cohort (22.8 ± 5.2 vs. 23.2 ± 3.3, *p* = 0.042) as well as the death cohort, had a smaller BMI (22.4 ± 3.5 vs. 23.3 ± 4.9, *p* < 0.001). This result was in accordance with the fact that frailty is a gradual, progressive process of deterioration which includes weight loss.^2^

Intriguingly, we found that majority of the variables that composed FI‐LAB did not increase the 5‐year mortality risk, except for PLT, CREA, ALT, and LDL‐C. Surprisingly, some variables even decreased the 5‐year mortality risk, including hematocrit, hemoglobin, TG, HDL‐C, and Alb. Nevertheless, patients with frailty had a significant higher increased risk of mortality compared to subjects without frailty. The Cox proportional hazard regression model was quite stable after compensating for age, gender, BMI, and other confounders. These results are in accordance with the theory of health‐related deficits as reflected by FI‐LAB.^16^, ^34^ In clinical practice, we should raise awareness regarding abnormal values of these laboratory variables.

Our study has several limitations. First, although we had the largest number of 2959 subjects, the distribution was quite irregular regarding location and tumor types, making generalisation of our findings difficult. Second, because enrolled individuals were only hospitalised cancer patients, there may be a survival bias. Third, other potential confounders, including income, education, exercise, and other chronic diseases were not adjusted for analysis. Fourth, this study did not provide data involving grip strength, speed of walking, and other variables part of the frailty phenotype, however, the frailty phenotype and frailty index are comparable, specifically when the cutoff point of the frailty index is set at 0.20–0.25.^19^, ^35^ Moreover, recent studies revealed that both frailty index and frailty phenotype can predict a 3‐year mortality risk.^36^ Finally, the predictive accuracy of the novel MCP algorithm was not satisfactorily high, due to the AUC being 0.691 (95% CI: 0.656–0.726) and 0.648 (95% CI: 0.613–0.684) in the training and validation sets.

Despite the limitations mentioned above, the novel MCP algorithm still encompasses a unique and practical function. By predicting the 5‐year MCP, it raised the patients’ awareness of frailty. In addition, gaining insight into risk factors could guide preventive strategies, particularly for potentially modifiable risk factors. For example, physical inactivity is recognized as one of the major contributing factors of frailty onset and progression, while physical exercise is known to preserve or improve frailty.^37^ Furthermore, several interventions in the clinical management of frailty are already well‐known such as lifestyle change, increasing physical activity, addition of nutritional foods and supplements, and cessation of any unnecessary medications.^4^, ^37^ However, more evidence‐based knowledge regarding effective intervention strategies, feasibility, and cost‐effectiveness of frailty are still needed. Besides, since individuals with frailty are able to dynamically transform, it is important to develop strategies for the delivery of care ranging across the continuum of frailty. Clinical care of cancer patients with frailty should also focus on maintaining functional independence and also other personal, individual‐centred, outcomes. The opportunities, challenges, and future directions we discussed give hope that the next generation of frailty management will improve the health outcomes of cancer patients and promote the quality of care.

## 5 CONCLUSION

In conclusion, we have successfully developed a novel MCP algorithm based on simple routine examinations with the ability to predict the 5‐year MCP. We also demonstrated that frailty is linked to an increased risk of mortality compared to those without frailty. Further studies using external multi‐institutional data sets are needed to confirm the current model and prospective cohort studies are needed to confirm the association between frailty and mortality.

## CONFLICT OF INTEREST

None of the authors have any competing interests.

## ETHICS APPROVAL

This observational, multi‐center, and hospital‐based prospective cohort study was registered with the Chinese Clinical Trial Registry (ChiCTR1800020329) and approved by the local ethical committees of all the participant hospitals, with formal written consent from every patient. This study was carried out in accordance with the declaration of Helsinki.

## CONSENT FOR PUBLICATION

All authors have given their consent for the publication of this article.

## Supporting information

Table S1

Table S2

## Data Availability

Tables (S1 and S2) and associated figure legends are provided as Supporting Information and are available online with the paper.
