# Supplementary material for: Prevalence of frailty and prediction of mortality in Chinese cancer patients using a frailty index‐based clinical algorithm—A multicentre study
Source: Cancer Med. 2021 Jul 28;10(18):6207–17. doi: 10.1002/cam4.4155 (PMC8446570; doi:10.1002/cam4.4155)
Supplement: Supplementary file 1 — Table S1 [file CAM4-10-6207-s001.docx]

**Supplementary Table**

**Supplementary Table 1, Routine blood laboratory variables used to construct the FI-LAB.**

| **Standard laboratory variables** | **Normal range or cutoff** | **HR (95% CI) for 5-year mortality** | **P-value** |
| --- | --- | --- | --- |
| White blood cells (number/L) | Men 4.0–9.2, women 3.7–9.2 | 1.003 (0.998–1.008) | 0.259 |
| Neutrophil (%) | 50–70 | 1.001 (1.00–1.002) | 0.069 |
| PLT (number/L) | 100–300 | 1.001 (1.00–1.002) | <0.001** |
| Red blood cells (number/L) | Men 4.1–5.7 women 3.7–5.1 | 0.999(0.972–1.028) | 0.965 |
| HGB | Men 131–172 women 113–151 | 0.990 (0.987–0.994) | <0.001** |
| HCT | Men 0.38–0.51 women 0.34–0.45 | 0.952(0.940–0.965) | <0.001** |
| MCV | Men 83.9–99.1 women 32.6–99.1 | 0.992 (0.980–1.004) | 0.205 |
| MCH | Men 27.8–33.8 women 26.9–33.3 | 0.980(0.951–1.009) | 0.173 |
| MCHC | Men 320–355 women 322–362 | 0.999 (0.994–1.004) | 0.676 |
| Blood sugar (mmol/L) | 3.9–6.1 | 1.028 (0.987–1.070) | 0.181 |
| TC | <5.18 | 0.990 (0.933–1.050) | 0.729 |
| TG | <1.70 | 0.863 (0.790–0.954) | 0.003 * |
| LDL-C | <3.37 | 1.120 (1.024–1.224) | 0.013* |
| HDL-C | ≧1.04 | 0.676 (0.534–0.854) | 0.001** |
| TBIL | 3.4–17.1 | 1.000 (0.997–1.002) | 0.837 |
| DBIL | <3.4 | 1.005 (0.997–1.012) | 0.207 |
| ALT | <55 | 1.002 (1.000–1.005) | 0.04* |
| Alb | 35–55 | 0.938 (0.925–0.950) | <0.001** |
| Glob | 9–34 | 1.003 (0.999–1.007) | 0.108 |
| BUN | 2.9–8.2 | 1.000 (1.000–1.000) | 0.115 |
| Creatinine | 53–140 | 1.009(1.006–1.012) | <0.001** |
| SUA (μmol/l) | 240–490 | 1.000 (0.999–1.001) | 0.904 |

HR, hazard risk; HGB, hemoglobin; HCT, hematocrit; MCV, mean corpuscular volume; MCH, mean corpuscular hemoglobin; MCHC, mean corpuscular hemoglobin concentration; TBil, total bilirubin; DBil, direct bilirubin; ALT, alanine transaminase; Alb: albumin; Glob, globulin; BUN, blood urea nitrogen;. *P < 0.05, **P < 0.01.
