# Supplementary material for: Prevalence of frailty and prediction of mortality in Chinese cancer patients using a frailty index‐based clinical algorithm—A multicentre study
Source: Cancer Med. 2021 Jul 28;10(18):6207–17. doi: 10.1002/cam4.4155 (PMC8446570; doi:10.1002/cam4.4155)
Supplement: Supplementary file 2 — Table S2 [file CAM4-10-6207-s002.docx]

**Supplementary Table 2, Characteristics of patients in the training and validation sets**

|  | training (n=966) | testing (n=1963) | P value |
| --- | --- | --- | --- |
| Age (years) | 56.0 ±10.3 | 55.7±12.4 | 0.509 |
| Male(%) | 42.1 | 63.9 | <0.001** |
| BMI (kg/m2) | 23.6±3.5 | 22.6±3.3 | <0.001** |
| Weight (kg) | 63.4±10.7 | 60.7±10.5 | <0.001** |
| Height (cm) | 163.8±7.8 | 163.7±7.8 | 0.705 |
| TG (mmol/l) | 1.6±1.2 | 1.5±1.0 | <0.001** |
| TC (mmol/l) | 4.7±1.6 | 4.8±1.3 | 0.860 |
| HDL-C(mmol/l) | 1.3±0.6 | 1.3±0.4 | 0.051 |
| LDL-C (mmol/l) | 2.7±1.2 | 3.1±0.9 | <0.001** |
| SUA (μmol/l) | 299.2±75.8 | 315.6±90.6 | <0.001** |
| Hypertension (%) | 16.6 | 17.7 | 0.450 |
| Diabetes (%) | 8.7 | 8.1 | 0.554 |
| Cardiovascular disease | 5.5 | 2.1 | <0.001** |
| Anemia | 2.7 | 0.8 | <0.001** |
| Chronic hepatitis | 8.6 | 3.8 | <0.001** |
| Frailty (%) | 54.9 | 54.0 | 0.635 |
